# Anticancer properties of soy-based tempe: A proposed opinion for future meal

**DOI:** 10.3389/fonc.2022.1054399

**Published:** 2022-10-24

**Authors:** Fahrul Nurkolis, Faqrizal Ria Qhabibi, Vincentius Mario Yusuf, Stanley Bulain, Ghevira Naila Praditya, Deogifta Graciani Lailossa, Msy Firyal Nadya Al Mahira, Eka Nugraha Prima, Tony Arjuna, Shilfiana Rahayu, William Ben Gunawan, Felicia Kartawidjajaputra, Dionysius Subali, Happy Kurnia Permatasari

**Affiliations:** ^1^ Department of Biological Sciences, Faculty of Sciences and Technology, State Islamic University of Sunan Kalijaga, Yogyakarta, Indonesia; ^2^ Medical Study Programme, Faculty of Medicine, Brawijaya University, Malang, Indonesia; ^3^ Department of Nutrition and Health, Faculty of Medicine, Public Health and Nursing, Universitas Gadjah Mada, Yogyakarta, Indonesia; ^4^ Department of Nutrition Science, Faculty of Medicine, Diponegoro University, Semarang, Indonesia; ^5^ Health and Nutrition Science Department, Nutrifood Research Center, PT Nutrifood Indonesia, Jakarta, Indonesia; ^6^ Faculty of Biotechnology, Atma Jaya Catholic University of Indonesia, Jakarta, Indonesia; ^7^ Biochemistry and Biomolecular, Faculty of Medicine, Brawijaya University, Malang, Indonesia

**Keywords:** tempe, anticancer, soy, molecular and cellular oncology, antioxidant, phytochemicals, peptides, functional food

## Introduction

Cancer is in the second rank of leading causes of mortality around the world in 2020, with around 10 million death cases, just behind heart disease, as well as approximately 250 million disability-adjusted life years (DALY) due to cancers (1). The most common form of deadly cancer is tracheal, bronchus, and lung cancer generally, and the number is greater for males. However, women also possess the threat of female-specific cancer, including breast (higher in women, but also happens in men), cervical, ovarian, and uterine cancer. This is also burdened by low-middle socioeconomic status (1, 2). In 2019, the number of new cancer incidents reached around 23.6 million cases, which increased from the 2010 incidence (18.7 million cases) (1). The risk factor includes smoking, unhealthy diets, and pollution, which increases the risks of developing cancer in the future (3). There have been several cancer treatment options in recent years, such as surgery, radiation, and chemotherapy. Only a third of cancer patients are estimated to be cured with surgery or radiation therapy, while in cancers that have spread, chemotherapy is needed as a systemic treatment option (4). These cancer treatment options can also cause major side effects and are relatively expensive. Thus, it is necessary to develop and find new medicines derived from natural ingredients, especially plant-based functional food, so that the availability of these drugs is abundant and the price is also relatively cheaper (4).

A diet high in soy has been associated with a lower prevalence of various types of cancer as well as improved treatment outcomes and lower recurrence rates after cancer diagnosis (5, 6). Tempe, a soy-based food originating from Indonesia, is reported to be capable of inhibiting proliferation and angiogenesis as well as triggering apoptosis in cancer cells (7). Soybean’s anticancer activities are related to phenolic compounds, saponins, and phytic acid as well as enzyme inhibitors such as trypsin and the Bowman-Birk inhibitors, but the most notable compounds are isoflavones, phytochemicals found in soybeans that can act as antioxidants and protect human cells from oxidative stress linked to cancer (8, 9). Genistein, a predominant isoflavone in soy, has been shown to inhibit cancer development, growth, and metastasis in animal models, particularly by modulating the genes related to cell cycle control and apoptosis (5). The fermentation process has been shown to increase the number of bioactive compounds and their activity in a food ingredient (10, 11). This is an interest in functional food development technologies and cemented food-based medicines, especially as promising future anticancer functional food candidates. One preclinical study highlighted the chemopreventive and chemotherapeutic potential of tempe (7).

There have been many review articles discussing tempe, but only in general its health benefits. There has been no review or opinion that summarizes the findings of the latest research that provides clarity on the benefits of tempe as an anticancer functional food. Therefore, the main purpose of this opinion article is to elaborate on the recent finding on the anticancer potential and activities of soy-based tempe. This opinion paper presents updated evidence based on literature reviews about the anticancer potential of soy-based tempe. The author also believes that by sharing this viewpoint, researchers would reinvent the creation of soy-based tempe food products and, of course, carry out more studies on their anticancer effectiveness.

## Soy-based tempe in general

Tempe is a fermented soybean food from Indonesia. Its distinctive taste and nutritional value make tempe one of the most consumed sources of protein for hundreds of years (12). Tempe consumption has the advantage of modulating the gut microbiota towards a healthier profile. This also shows that the consumption of tempe provides a healthier metabolic status. To maintain the composition of the beneficial gut microbiota, the consumption of dietary fiber, paraprobiotics, and probiotics from Soy-based tempe is highly recommended (13). Besides being the main protein source, soy-based tempe has been developed as a carbohydrate substitute in the form of flour. It has been found that tempe flour-based products have higher flavonoid content than wheat-based products (14). Because of its high glutamic acid and protein content, overripe tempe is utilized both as a source of umami flavor and protein in porridge for children under five years old to help overcome children’s malnutrition (15). Recent research has also proven that tempe application as yogurt has higher antioxidant, carbohydrate, protein, and lipid content compared with plain cow milk yogurt, thus it is being mass-produced (16). Furthermore, the bioactive peptides synthesized from soy-based tempe fermentation can be utilized as anti-hypertensive, antioxidant, anti-diabetic, and anticancer agents (17). Because of the various benefits of soy-based tempe, it leaves more room to explore the potential of tempe health properties, specifically as an anticancer agent.

## Anticancer activity and properties of soy-based tempe

**Figure 1:**
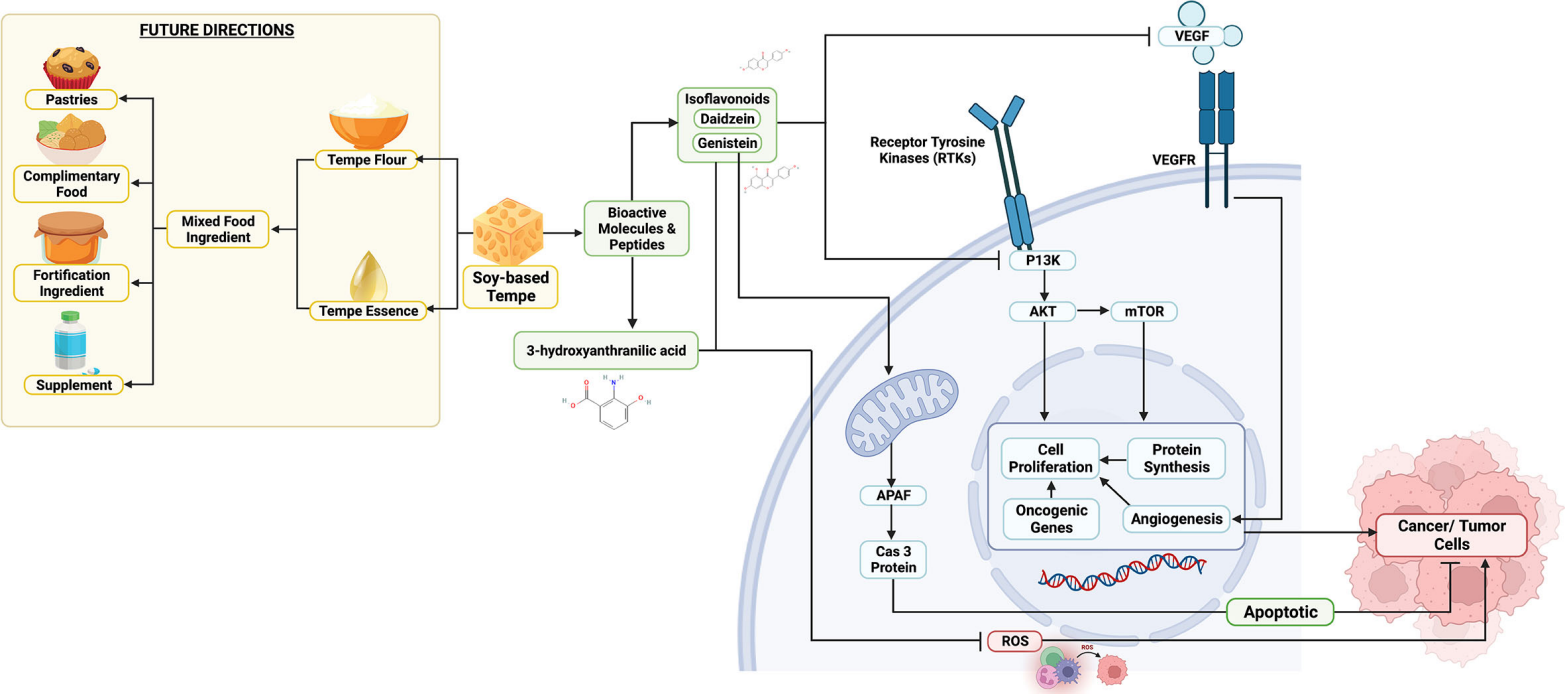
Predicted Mechanism of Anticancer Properties from Soy-Based Tempe and Their Future Meal Product Innovation.

Tempe is a soy-based fermented food that contains bioactive peptides such as calmodulin-dependent cyclic nucleotide phosphodiesterase (CaMPDE) inhibitors which are also rich in isoflavones and their derivatives such as genistein and daidzein (7, 18). Bio-functionality tests on bioactive chemicals derived from tempe have been shown to have anticancer or antitumor action. Anticancer mechanisms mediated by bioactive chemicals in tempe include cancer cell proliferation inhibition, angiogenesis inhibition, antioxidant properties, and induction of cancer cell apoptosis (7, 18). Inhibition of the cancer cell proliferation mechanism by isoflavones happened due to the inactivation of the phosphoinositol 3-kinase (PI3K)/Akt/mTOR pathway (19). When cancer cells are exposed to genistein, an isoflavone derivative, the expression level of the PI3K protein decreases, which has the key role of activating numerous proteins for cell growth and aberrant cell proliferation induction (18–20). Angiogenesis was also inhibited by the presence of genistein, which can reduce either the expression or excretion of vascular endothelial growth factor (VEGF) (7, 21). The VEGF protein stimulates cell proliferation by causing the development of new blood vessels, a process known as angiogenesis. Angiogenesis plays an important role in cancer cell proliferation and metastasis because it supplies adequate oxygen and nutrients (7). Therefore, inhibition of angiogenesis in cancer tissue is an important mechanism in suppressing cancer progression. The antioxidant activity of tempe is due to the presence of isoflavones and 3-hydroxyanthranilic acid which have excellent free radical and superoxide scavenging abilities which have been proven through the DPPH (2,2-diphenyl-1-picrylhydrazyl) assay (22). Tempe’s antioxidant activity helps protect healthy cells from genetic material damage, which can develop into oncogenic genes. Genistein is also able to induce apoptosis through its effect on oxidative phosphorylation in mitochondria due to the inactivation of the anti-apoptotic Bcl-2 gene (7). Because the rise of Bcl-2 expression in cancer cells is not accompanied by an increase in Cas-3 protein, Bcl-2 acts as an anti-apoptotic protein. Meanwhile, genistein promotes the co-expression of Bcl-2 and Cas-3 proteins through the apoptotic protease activating factor (APAF) (**Figure 1**). An increase in the amount of Cas-3 protein which acts as an effector caspase will trigger apoptosis in cancer cells (23).

## Future direction and implications of soy-based tempe as a functional meal

Soy-based tempe – in addition to having promising potential as a functional food anticancer – also has the potential for product development into a varied and innovative healthy meal (**Figure 1**). Tempe can be used as a mixed food ingredient (MFI). The latest research combined tempe flour with eel flour which has bioactive peptides, nutrition content, and scavenging of potential anticancer free radicals (24, 25). Recent research has also combined tempe flour with algae and has succeeded in determining the potential antioxidant effects, one of which is inhibiting cancer-inducing free radicals (26, 27). Then, tempe flour can also be used as a healthy meal program for toddlers, children, and adults. An example of a food product with tempe essences is complementary food breast milk (breast milk) (28). This is one of the potential efforts from an early age to mediate the incidence of cancer. In addition, tempe can also be used as an ingredient to fortify biscuits for the prevention of anemia (29). This is a special interest for vegetarians or those who are on a plant-based diet because tempe is a vegetable superfood and source of Fe (Iron) which is useful for vegans who tend to have minimal Fe intake. In addition, a study shows that a vegetarian diet seems to protect against cancer (30). This reveals the importance of implementing a soy-based tempe-based healthy meal which can potentially minimize the prevalence of cancer through the mechanisms that have been described in the previous sub and **Figure 1**.

## Discussion

Tempe primarily contains bioactive peptides and isoflavones which could promote health and prevent cancer due to their antioxidant activities, cancer cell apoptosis, and cancer cell proliferation inhibition activities (7, 18). The role of an antioxidant in cancer therapy is to counterbalance the production of reactive oxygen species (ROS) and their mediated injuries in the affected molecules (DNA, lipids, or proteins) (31). On the other side, a dietary intervention strategy that incorporates food with antioxidant properties also brings more beneficial outcomes on health status (32). Therefore, this opinion supports the consumption of tempe as a dietary antioxidant to promote health due to its anticancer potential, which was also highlighted by Wu et al., 2017 (31). Various applications and processing of tempe to be a functional food had also been described. The combinations of tempe with other ingredients brought more beneficial effects on health, nutrient composition, and more acceptable food products (24–27, 29). Utilizing tempe as a flour product also helps to increase the shelf-life period of the products (33). However, it is important to preserve the fresh probiotic properties of tempe, especially in larger industries or production scales since they have health-beneficial properties (34). Tempe supplementation was also known as a paraprobiotics agent which is capable of modulating gut microbiota, enhancing immunity, and alleviating inflammation (13, 35). Next to that, the importance of foodomics may be crucial for maximizing the health potential of tempe products. Foodomics is a new approach that utilizes the “omics” method in food and nutrition studies to bring forth the optimization of human health and well-being (36). As a fermentation product, tempe produces many bioactive compounds and secondary metabolites which could be further analyzed using the foodomics approach to find the specific compound needed for specific health issues. A metabolomic study found that using different starter cultures of tempe will result in different metabolite profiles of tempe products (12). On the other hand, a proteomic study dove deeper into the antihypertensive, antidiabetic, antioxidant, and antitumor activities of the bioactive peptides from tempe (18). Therefore, this opinion paper encourages more research that brings forth the innovation of tempe as a functional food product or many deeper types of research that utilize the foodomics to draw out tempe best potential as an anticancer, Indonesian functional food.

## Author contributions

FN, FQ, VY, SB, GP, DL, MM, EP, TA, SR, HP, WG, FK, and DS, contributed to the conception and design of opinion studies and drafted manuscripts in advance. FN, FQ, and HP edited, revised, and approved the final version of the submitted manuscript. All authors contributed to the article and approved the submitted version.

## Acknowledgments

We offer a great thank you to the Chairman of the Indonesian Association of Clinical Nutrition Physicians, namely Professor *Nurpudji Astuti Taslim*, MD., MPH., PhD., Sp.GK(K) and Professor *Hardinsyah*, Ph.D. [President of the Federation of Asian Nutrition Societies (FANS)] who have reviewed and provided suggestions, as well as input on the draft of this opinion article. We also thank Dr. *Nelly Mayulu*, MD., who has given her views on the nutrition dan functional food programs that are important to do now and in the future for anticancer treatments. We also thank the *Nutrifood Research Center* (PT. Nutrifood Indonesia) for providing support to the author in this publication. All of these mentioned have no conflict of interest.

## Conflict of interest

Author FK was employed by the company PT. Nutrifood Indonesia.

The remaining authors declare that the study or opinion article was conducted in the absence of any commercial or financial relationships that could be construed as a potential conflict of interest.​

## Publisher’s note

All claims expressed in this article are solely those of the authors and do not necessarily represent those of their affiliated organizations, or those of the publisher, the editors and the reviewers. Any product that may be evaluated in this article, or claim that may be made by its manufacturer, is not guaranteed or endorsed by the publisher.
